# The role of large mammalian herbivores in shaping and maintaining soil microbial communities of natural mineral licks: A case study on sika deer at the firebreak adjacent to the Sino‐Russian border

**DOI:** 10.1002/ece3.10878

**Published:** 2024-02-01

**Authors:** Peiying Wen, Feng Wu, Lei Bao, Tianming Wang, Jianping Ge, Hongfang Wang

**Affiliations:** ^1^ National Forestry and Grassland Administration Key Laboratory for Conservation Ecology in the Northeast Tiger and Leopard National Park Beijing China; ^2^ Northeast Tiger and Leopard Biodiversity National Observation and Research Station Beijing China; ^3^ Ministry of Education Key Laboratory for Biodiversity and Ecological Engineering, College of Life Sciences Beijing Normal University Beijing China

**Keywords:** camera traps, *Cervus nippon*, natural mineral licks, northeast Tiger and Leopard National Park, soil microbes, soil physicochemical properties

## Abstract

Mineral licks are indispensable habitats to the life history of large mammal herbivores (LMH). Geophagy at licks may provide the necessary minerals for LMH, while LMH may be ecosystem engineers of licks by altering vegetation cover and soil physicochemical properties (SPCP). However, the precise relationship between the LMH and licks remains unclear. To clarify the geophagy function of licks for LMH and their influence on soil at licks, we recorded visitation patterns of sika deer around licks and compared SPCP and microbial communities with the surrounding matrix in a firebreak adjacent to the Sino‐Russian border. Our study indirectly supports the “sodium supplementation” hypothesis. Proofs included (1) a significantly higher sodium, iron, and aluminum contents than the matrix, while lower carbon, nitrogen, and moisture contents; (2) significantly higher deer visitation during sodium‐demand season (growing season), along with an avoidance of licks with high iron contents, which is toxic when overdose. The microbes at the licks differed from those at the matrix, mainly driven by low soil carbon and nitrogen and altered biogeochemical cycles. The microbial communities of licks are vulnerable because of their unstable state and susceptibility to SPCP changes. Structural equation modeling (SEM) clearly showed a much stronger indirect effect of deer on microbes at licks than at the matrix, especially for bacteria. Multiple deer behaviors at licks, such as grazing, trampling, and excretion, can indirectly shape and stabilize microbes by altering carbon and nitrogen input. Our study is the first to characterize soil microbial communities at mineral licks and demonstrate the processes by which LMH shapes those communities. More studies are required to establish a general relationship between the LMH and licks to promote the conservation of natural licks for wildlife.

## Graphical abstract

The geophagy of sika deer at natural mineral licks mainly involves supplementation with sodium during the growing season. Licks are vulnerable due to their unstable soil microbes and susceptibility to carbon and nitrogen perturbation. Sika deer play critical roles in shaping and stabilizing microbes at licks through multiple behaviors such as grazing, trampling, and excretion.

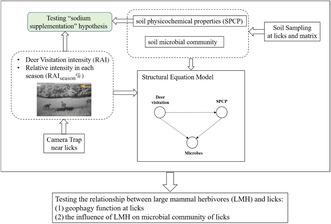

## 1 INTRODUCTION

Natural mineral licks are usually bare rock or soil that are rich in minerals (Kreulen, 1985; Matsubayashi et al., 2006; Molina et al., 2014). Geophagy or soil ingestion at mineral licks is a widespread and important behavior in a large number of herbivores (He et al., 2022; Kroesen et al., 2020; Lavelle et al., 2014; Li et al., 2019; Matsubayashi et al., 2006). Although geophagy has been considered a tropical‐subtropical phenomenon (Ghanem & Voigt, 2014; Gilmore et al., 2020; Link et al., 2011; Matsubayashi et al., 2006; Molina et al., 2014; Stephenson et al., 2011; Weeks, 1978; Weir, 1969), the behavior is widespread in northern ecosystems as well as in semi‐arid regions (Klein & Thing, 1989; Kroesen et al., 2020; Lavelle et al., 2014; Panichev et al., 2012). Multiple hypotheses have been proposed to explain the prevalence of geophagy, with LMH seeking sodium supplementation at licks being the most commonly accepted (Atwood & Weeks, 2003; Duvall et al., 2023; Krishnamani & Mahaney, 2000; Weeks & Kirkpatrick, 1976). In addition to sodium, mineral licks likely supply other nutrients such as calcium and magnesium, aiding in detoxifying minor plant compounds, and alleviate gastrointestinal disorders (Klaus et al., 1998; Monaco et al., 2019). The function of geophagy at mineral licks varies greatly depending on the herbivore species, age, sex, and the type of lick (Ghanem et al., 2013; Molina et al., 2014; Panichev et al., 2017; Tracy & McNaughton, 1995). Owing to the uncertainty of the geophagy function at natural mineral licks, non‐natural licks created by human activity (such as road construction) or artificial salt sources cannot always replace natural mineral licks successfully (Simpson et al., 2020). Therefore, more studies in various climate regions are needed to facilitate general understanding of the geophagy function of natural mineral licks (hereafter called “licks”).

While generalizable patterns on the functional roles of geophagy have not yet been identified, licks are indisputably important habitats for large mammalian herbivores (LMH). Indeed, maintenance of licks is likely dependent on LMH activities like grazing, trampling, digging, and excretion (Ghanem & Voigt, 2014; Weir, 1969). Hence, LMH species likely act as ecosystem engineers by developing and maintaining licks. One of the mechanisms by which they do so may be the changes their activities make to the licks' vegetation cover and soil properties, likely producing changes to the licks' soil microbial communities. However, no studies to date have investigated either lick microbial communities or LMH species' influences on those communities.

In this study, multiple data types were collected from licks to clarify the geophagy function of licks for a LMH species, sika deer, in a temperate region as well as the deer influence on soil microbes. Data types collected were: (1) camera trap data near the licks, which was used to generate the visitation intensity of deer to licks; (2) soil physicochemical properties (SPCP) of licks and the surrounding background (hereafter called the “matrix”), including organic carbon content (SOC), total nitrogen content (N), soil moisture (SMM), and 10 mineral content indices (P, K, Na, Ca, Mg, Al, Fe, Mn, Cu and Zn); (3) soil microbial community data of licks and matrix, generated using DNA metabarcoding. By utilizing these data, we tested the “sodium supplementation” hypothesis. If deer visited the licks to supplement sodium, we would expect (1) higher Na content at licks than at the matrix and (2) higher intensity of deer visitation during the high sodium demand season. According to existing studies, plants usually contain insufficient sodium, which may decrease as the growing season progresses (Morris et al., 1980). Additionally, deer may experience increased demand for sodium during gestation and lactation (Atwood & Weeks, 2002; Pletscher, 1987). The gestation period for sika deer is usually from September to October and May to June, and lactation encompasses the entire growing season and usually stops at the end of summer (Sadleir, 1980). Hence, we expect sika deer to visit licks more intensively during the growing season; (3) Licks with higher Na content attract more deer visitation during the high sodium demand season. Sodium content may represent lick quality for LMH, and high‐quality habitats are expected to attract larger deer populations (Lavelle et al., 2014; Velamazán et al., 2018).

In addition, we characterized soil microbial profiles. Owing to vegetation cover differences, we firstly expect the main driving force of the microbial community difference between licks and the matrix to be SOC and N. Secondly, the bare soil status at licks, as detailed above, usually leads to the features of licks being unstable. Hence, we expect the microbes at the licks to be more unstable than those at the matrix. Several indices can be used to characterize the stability of the microbial community: (1) the relative abundance ratio of bacteria to fungi (BFratio). Existing studies suggest that soil fungal communities are more stable than bacterial communities, whereas bacteria respond to environmental changes more quickly than fungi (de Vries et al., 2018). Hence, bacteria at the licks are expected to increase in abundance and diversity more significantly than fungi; (2) AVD is another common index, reflecting community stability (Wang et al., 2022; Xun et al., 2021). AVD is the average variation degree of the community, with a higher AVD value representing greater randomness and, thus, lower stability.

Lastly, we aimed to understand how sika deer influence microbes at licks. Firstly, because sika deer graze, trample, and excrete at licks, we expected sika deer to influence microbes directly or indirectly by altering the input of SOC or N. Secondly, as mentioned above, the LMH are reported to be able to maintain the stability of licks (Ghanem & Voigt, 2014; Weir, 1969). Hence, we also expect that LMH activity can increase the stability of microbial communities and that such an effect may only be significant at licks but not at the matrix. A structural equation model (SEM) was employed to uncover the path and strength of the influence of sika deer on microbes at the licks and matrix.

## 2 MATERIALS AND METHODS

### 2.1 Study area

**FIGURE 1:**
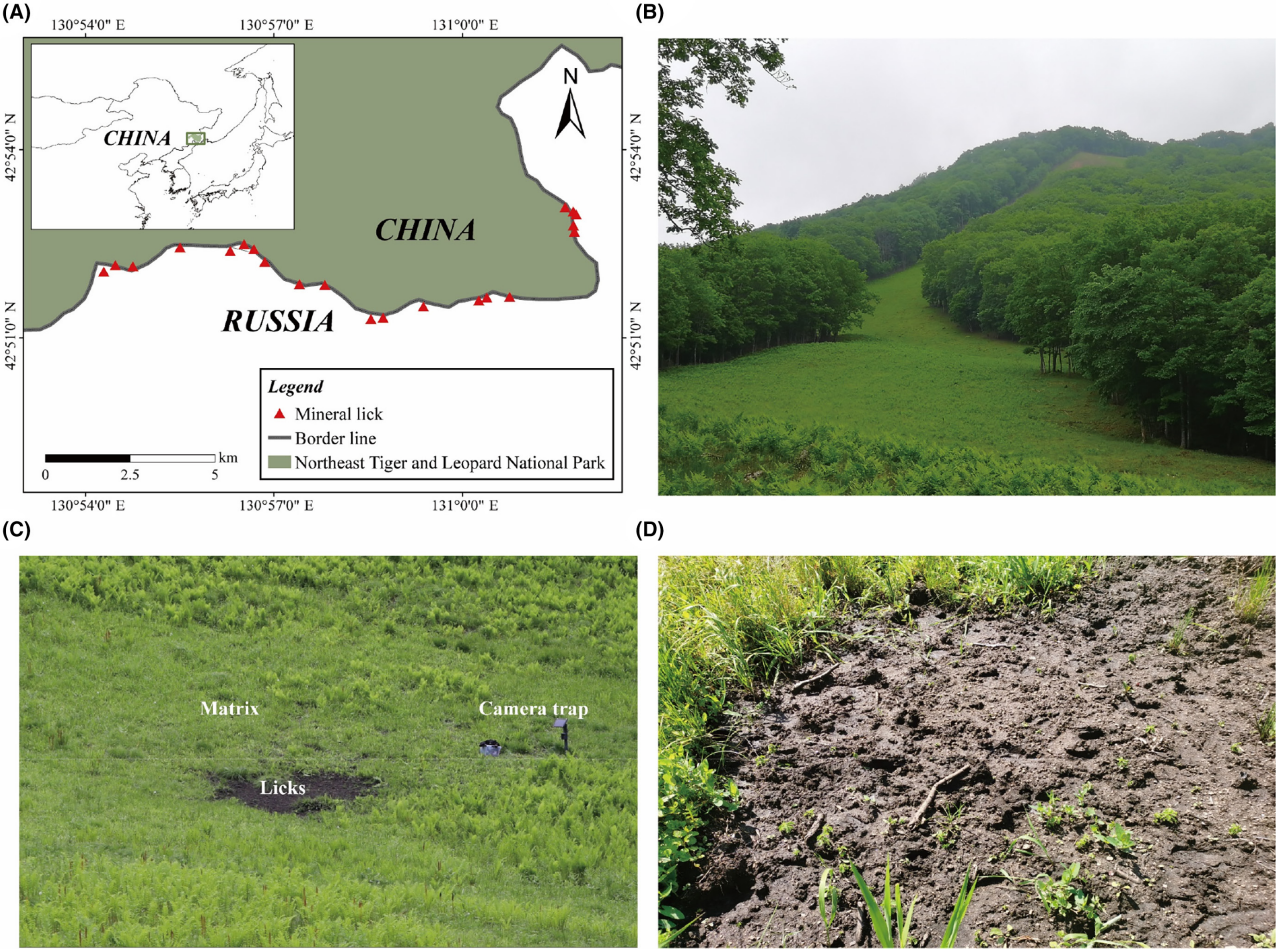
(A) Spatial location of the studied 21 at the firebreak near the Sino‐Russian border. The inset map shows the location of the Northeast Tiger and Leopard National Park, where our study was taken. Multiple photographs show (B) the firebreak landscape; (C) position of lick, matrix, and camera trap in an example lick; (D) a close view of a lick with bare soil status and footprint of sika deer.

Our study area is located in the Northeast Tiger and Leopard National Park, located in Northeast China (129°5′0″–131°18′48″ E, 42°31′06″–44°14′49″ N, Figure 1A), with a coniferous and deciduous broadleaf mixed forests as its dominant vegetation.

Sika deer (*Cervus nippon*) are among the main LMH species in the cool temperate forest ecosystems. Their populations are mainly restricted to the southeast corner of the National Park (Figure 1A), especially at the firebreak adjacent to the Sino‐Russian border (Wang, Feng, et al., 2016). There are other LMH in the National Park, such as *Capreolus pygargus*, *Hydropotes inermis*, *Cervus canadensis*, and *Moschus moschiferus*; however, monitoring data shows that only sika deer are distributed near and at the 50‐meter‐wide firebreak at the border (Wang, Feng, et al., 2016). All plant tissues (>0.5 m) within the firebreak are cut every September to control the spread of fire. Under regular mowing, the firebreak is transformed into a grassland landscape consisting of continuous and low grasses, with *Gramineae*, *Cyperaceae*, and *Liliaceae* as the three most dominant families (Figure 1B). Licks are easily identified in firebreaks because of their distinct lack of vegetation cover and noticeable traces of herbivore visitation: bare soils with clear boundaries to distinguish them from the matrix usually characterize the licks (Figure 1C,D). Based on data obtained from camera traps near licks, sika deer exhibit different behaviors at licks and matrix. In addition to geophagy, the most striking behavior that sika deer exhibit at licks is hopping, a behavior that is rare outside licks (personal observation, Video S1).

Approximately 35 licks, ranging in size from 4 to 15 m^2^, were found within a 16 km long section of the firebreak. Twenty‐one easily‐accessed licks (~60%) were selected for subsequent analyses. The average Euclidean distance between adjacent licks was 400 m, ranging from 109 to 1094 m (Figure 1A). Although the Euclidean distance was small for some licks, the steep slope between these licks restricted free sika deer movement between licks, which generated no significant spatial autocorrelated pattern for deer visitation (data not shown).

### 2.2 Camera traps and deer visitation intensity

Unbaited camera (LTl‐6511‐4G, Shenzhen, China) were purposefully placed near 21 licks to monitor sika deer visitations to the licks since August 2020 (Figure 1C). All camera traps were pointed at the whole or part of licks, about 1–5 m from the licks, depending on the size of licks. The cameras, about 80–100 cm above the ground, were powered using solar panels and maintained at least every 2 months for continuous monitoring. The cameras were programmed to take videos 24 h/day with a 1‐min interval between consecutive 10 s videos.

To reduce workload, we only dealt with the activity data of sika deer in October 2020 (Fall) and January (Winter), May (Spring), and August 2021 (Summer) to represent the activity pattern of the four seasons. Because deer may visit licks frequently when they require geophagy, we used deer visitation intensity to characterize the intensity of geophagy. The number of independent events captured using camera traps during a certain period is a common way to reflect intensity of animal visitation (Carbone et al., 2001; Griffiths et al., 2023; Rovero & Marshall, 2009; Tanwar et al., 2021). We calculated deer visitation intensity for each camera trap near the 21 licks as follows: (1) the number of independent deer visitation events was determined for each sampling month (relative abundance index, hereafter “RAI_season_,” the season was “spring,” “summer,” “fall” and “winter” mentioned as above). Independent events were defined as two captures with time intervals longer than 30 min, a common criterion in such calculations (O'Brien et al., 2003; Wang, Feng, et al., 2016). Only cameras active in all four seasons were involved in the following calculation; (2) the overall deer visitation intensity of each lick (hereafter “RAI”) was the sum of four RAI_season_: $\mathrm{RAI}=\sum_{\text{season}}^{4}{\mathrm{RAI}}_{\text{season}}$. Since six cameras failed to record any videos in one of the four seasons, the final sample size for RAI was 15.

### 2.3 Soil sample collection and SPCP measurements

For each of the 21 licks, soil samples were collected using the five‐point collection method (Zhang, 2011) from October 3 to 5, 2020: one from each lick and one from the nearby matrix. Since complex biotic or abiotic factors can cause heterogeneity of SPCP and soil microbes (Jansson & Hofmockel, 2020; Kuzyakov & Blagodatskaya, 2015), to uncover the exact differences of licks and matrix, we collected the matrix samples approximately 10 m from the licks. Existing studies suggest that comparing licks with adjacent matrix is sufficient to detect SPCP differences between licks and the matrix (Griffiths et al., 2023; Holl & Bleich, 1987; Monaco et al., 2019). Moreover, biogeochemical processes in mounds shaped by termites and surrounding matrix also support dramatic change within small spatial scales (Baker et al., 2020; Fox‐Dobbs et al., 2010). Hence, we restricted our matrix samples to within 10 m of the edges of licks (Figure 1C).

Mahaney and Krishnamani (2003) suggested that LMH in tropical and subtropical regions avoid geophagy at the topsoil. According to our observations, sika deer generally ingest topsoil at licks (Video S2). In addition, trampling and hopping may directly influence soil microbes of the topsoil. Hence, only the topsoil (0–10 cm) with the ground cover removed was sampled. Disposable gloves and shovel covers were used to minimize contamination. After collection, all samples were immediately placed in an icebox and brought back to the laboratory. Each sample was divided into two parts. One portion was stored in a 15 mL centrifuge tube for microbe experiments at −80°C until DNA extraction. The other portion was stored at room temperature in a resealable bag for SPCP analysis.

Soil moisture content (SMM) was measured using the drying method. Sub‐samples were dried in a thermostatic drying chamber at 105°C for 12 h. The remaining soil samples were dried naturally, and soil particles bigger than 0.149 mm were removed by sieving prior to analysis (Zhang, 2011). SOC was measured using the potassium dichromate oxidation‐external heating method (Nelson & Sommers, 1996). N was measured using an automatic Kjeldahl nitrogen analyzer (UDK152, VELP Scientifica Srl, Usmate, Italy) (ISO, 1995). Ten mineral content indices (P, K, Na, Ca, Mg, Al, Fe, Mn, Cu, and Zn) were measured using an ICP atomic emission spectrometer (IRIS Intrepid II XSP; Thermo Fisher Scientific, Waltham, MA, USA) (ISO, 2008).

### 2.4 DNA metabarcoding and bioinformatics analysis

DNA metabarcoding was used to characterize the microbial community of each soil sample. DNA was extracted using a PowerSoil DNA Isolation Kit (MO BIO Laboratories, Inc., Carlsbad, CA, USA). The quality of the DNA extracts was assessed using a Micro UV Spectrophotometer (NanoDrop ND‐2000, Thermo Fisher Scientific), and DNA integrity was detected by agarose gel electrophoresis. The V3‐V4 region of bacterial 16S rRNA was amplified by PCR using the primers 338F (5′‐ACTCCTACGGGAGGCAGCAG‐3′) and 806R (5′‐GGACTACHVGGGTWTCTAAT‐3′). The ITS1 region of fungal internal transcribed spacer (ITS) rRNA was amplified by PCR using the primers ITS1F (5′‐CTTGGTCATTTAGAGGAAGTAA‐3′) and ITS2R (5′‐GCTGCGTTCTTCATCGATGC‐3′). Amplification was performed using a GeneAmp 9700 thermocycler (Applied Biosystems). The PCR products were analyzed using a 2% agarose gel, ensuring that the size of the PCR product was correct and that the concentration was appropriate. Sequencing was performed by Shanghai Majorbio Bio‐pharm Technology Co. Ltd. (Beijing, China) using an Illumina MiSeq platform (San Diego, CA, USA).

Fastp (version 0.19.6; https://github.com/OpenGene/fastp) was used to remove low‐quality sequences shorter than 50 bp. The average lengths of the bacterial 16S rRNA and fungal ITS sequences obtained were 416 and 233 bp, respectively. UPARSE software (version 7.0, http://www.drive5.com/uparse/) was used to cluster operational taxonomic units (OTUs) with a similarity threshold equal to or higher than 97%. To identify the classification status of each OTU, we used the Silva database (version 119, http://www.arb‐silva.de) for bacterial 16S rRNA sequences and the Unite database (version 6.0, http://unite.ut.ee/index.php) for fungal ITS sequences.

### 2.5 Testing the “sodium supplementation” hypothesis

To compare the SPCP differences between licks and the matrix and to test whether licks had higher sodium levels, Wilcoxon rank sum test was applied to the 13 SPCP indices. The Spearman's correlation between the SPCP values was calculated to determine the extent of co‐occurrence between the SPCP indices.

To test whether sika deer visit licks more intensively during the growing season, we compared the deer visitation intensity between seasons using the Kruskal–Wallis rank sum test, with “Benjamini–Hochberg” as the *p*‐value correcting method.

To determine whether licks with higher minerals can attract more deer visitation during the high‐demand season, weighted linear regression models were performed with *rma* function in package *metafor* v4.4‐0 (Viechtbauer, 2010). Since licks with lower detection rate (small RAI) were expected to have bigger variance, each lick was weighted by the overall deer visitation intensity (RAI) during regression modeling, with licks having larger RAI weighted more in the models. Only seasons with intensive deer visitation, which were expected to have high demand for geophagy, were involved in the regression modeling. Due to the small sample size (*n* = 15), we conducted the regression models for each RAI_season_ with each of the 10 minerals at licks independently.

All the above analysis were conducted in R 4.3.1 (R Core Team, 2022).

### 2.6 Characterizing the profile of microbes at licks

The microbial community structure was characterized using three indices: diversity (Shannon), variation (AVD), and the relative abundance ratio of bacteria to fungi (BFratio). Shannon index considers both the richness and evenness of the community, with a higher value representing higher diversity. Shannon and AVD were calculated independently for the bacterial and fungal communities. Shannon analysis was conducted using Mothur (version 1.30.2; https://www.mothur.org/wiki/Download_mothur). AVD was calculated according to Xun et al. (2021). All three indices were calculated independently for the licks and matrix, and their differences were tested using the Wilcoxon rank‐sum test.

Differences in microbial composition between the licks and the matrix were compared using analysis of similarity (ANOSIM). ANOSIM was conducted using the package *vegan* (Oksanen et al., 2020). To detect microbes with significant differences between the licks and the matrix, we performed LEfSe analysis (Galaxy, version 1.0.0; http://huttenhower.sph.harvard.edu/galaxy), and the parameter settings were LDA > 3 and *p* < .05.

Redundancy analysis (RDA) and canonical correspondence analysis (CCA) were applied to analyze the relationship between microbial composition and SPCP, depending on the length of the first ordination axis (threshold value:3.5) (Wang, Wang, et al., 2016). Only SPCP indices with significant differences between licks and the matrix were included in the RDA/CCA to focus on factors driving the profile of microbes at licks. Since SOC and N were strongly associated, as shown in our results (licks: Spearman's ρ = 0.96, *p* < .001; matrix: Spearman's ρ = 0.84, *p* < .001; Table S2), N was not included in the analysis. RDA/CCA were conducted using the “vegan” package.

The functions of the identified bacteria and fungi were predicted by mapping the identified taxa to existing databases to test whether differences in vegetation could drive divergence in biogeochemical processes. FAPROTAX is a database that maps prokaryotic clades to establish ecologically relevant functions, such as carbon and nitrogen cycles (Louca et al., 2016; Sansupa et al., 2021). Bacterial OTUs were matched to the FAPROTAX database and annotated using functional information. The number of OTUs for each detected function and relative abundance were determined separately for the licks and the matrix. The Wilcoxon rank‐sum test was used to compare the relative abundance of each function between the licks and matrix. FUNguild is a tool that assigns fungal clades to specific ecological guilds or trophic modes (Nguyen et al., 2016). Only OTUs with significant differences between the licks and the matrix in LEfSe were applied to FUNguild analysis.

### 2.7 Analyzing the effect of deer visitation on soil microbial community structure

**FIGURE 2:**
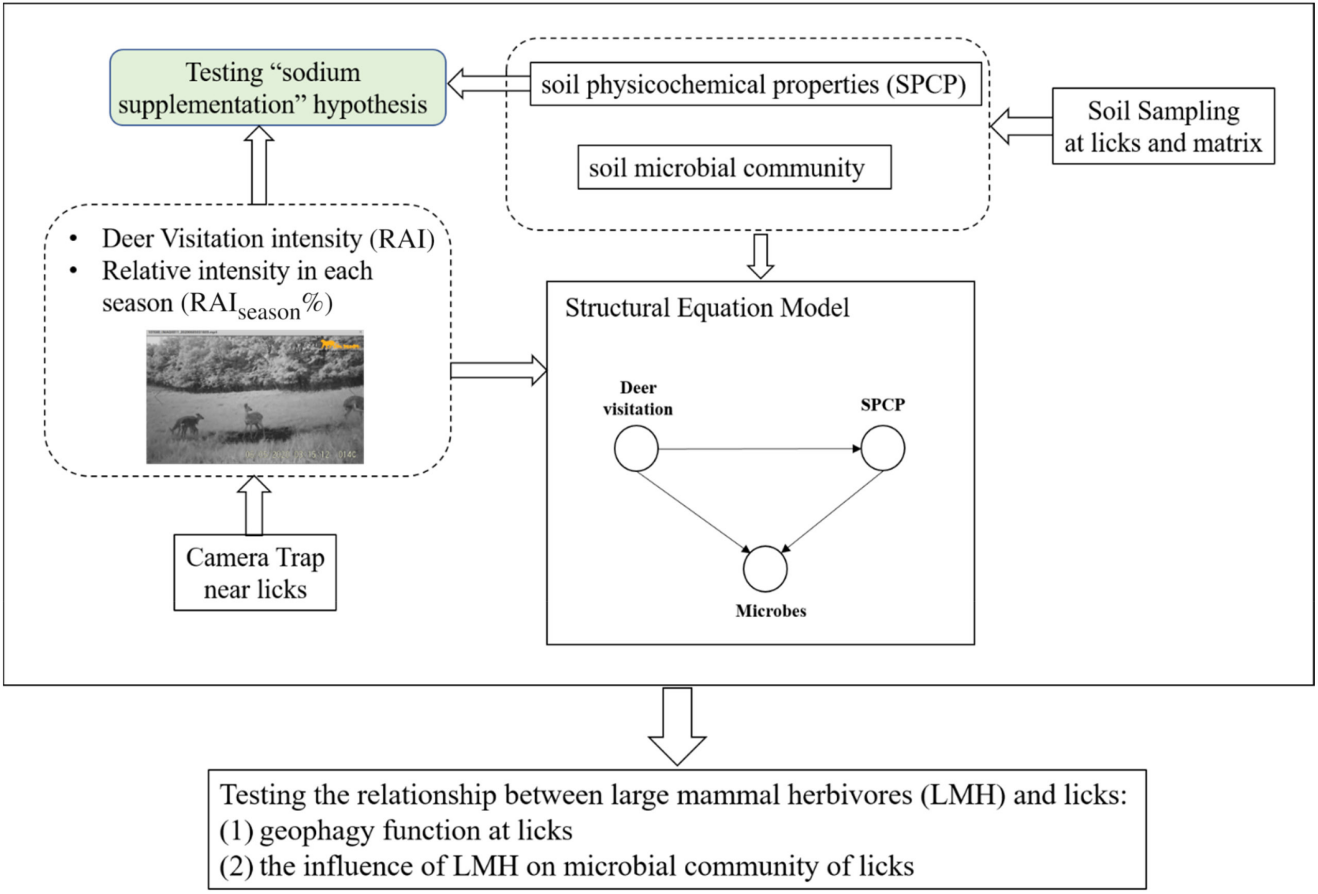
A flowchart shows the design and main purpose of this study.

A SEM was used to test the direct effect of deer visitation on microbes or the indirect effect through SPCP (Figure 2). However, the sample size in this study (*n*_lick_ = *n*_matrix_ = 15) was small to meet the requirements of the traditional covariance‐based SEM. To use the SEM model with a small sample size: (1) we opted for a partial least squares structural equation model (PLS‐SEM). PLS‐SEM is a variance‐based SEM that efficiently estimates path models with latent variables and their relationships within a limited sample size (Cassel et al., 1999; Hair et al., 2019). It makes no distributional assumption and has good statistical power in identifying potential relationships (Cepeda‐Carrion et al., 2019; Hair et al., 2017); (2) To reduce the complexity of the model, only manifest (directly measurable) variables were involved. Deer visitation was measured using the manifest variable, RAI. There were at least 13 variables in our study able to measure latent variable SPCP. However, only SOC was used in the PLS‐SEM calculation, as SOC and N are considered the most important determinants of soil microbes (Ni et al., 2021; Yang et al., 2020), and they are potentially strongly influenced by LMH (He et al., 2019; Millett & Edmondson, 2015; Xun et al., 2018). N was removed because of its strong correlation with SOC (Table S2). Five manifest variables were used to measure the community structure of microbes (Shannon, AVD of bacteria and fungi, and BFratio). Hence, five separate path models were calculated for each manifested microbial variable.

According to our results, several manifested variables were significantly different between licks and matrix (Table 1), which could not satisfy the prerequisite (measurement invariance) for pooling data of the two groups (Henseler et al., 2016). Multigroup analysis (MGA) can reflect the heterogeneity in structural models between licks and matrix (Sarstedt et al., 2011). Hence, MGA in PLS‐SEM was conducted using SmartPLS software (version 4, SmartPLS GmbH, Oststeinbek, Germany), and bootstrap was utilized to test the differences in path coefficients between the licks and the matrix.

**TABLE 1:** Soil physicochemical properties (SPCP) differences between licks and matrix.

Soil physicochemical properties	Licks	Matrix	Wilcoxon rank sum test	
			*W*‐value	*p*‐value
**SOC (g/kg)**	97.17 ± 10.62	152.34 ± 6.03	360	**.000**
**N (g/kg)**	4.69 ± 0.52	7.16 ± 0.31	351	**.001**
**SMM (%)**	11.92 ± 1.01	16.51 ± 1.15	330	**.005**
**Na (g/kg)**	8.86 ± 0.37	7.82 ± 0.14	133	**.028**
**Fe (g/kg)**	35.86 ± 1.56	31.28 ± 0.92	138.5	**.040**
**Al (g/kg)**	45.22 ± 1.67	40.83 ± 0.57	86	**.000** **
P (g/kg)	0.88 ± 0.08	1.05 ± 0.07	266	.261
K (g/kg)	15.51 ± 0.66	14.87 ± 0.39	165.5	.170
Ca (g/kg)	10.35 ± 1.26	10.02 ± 0.75	229	.842
Mg (g/kg)	7.50 ± 1.10	6.25 ± 0.49	219	.980
Cu (g/kg)	0.012 ± 0.001	0.014 ± 0.001	270.5	.210
Zn (g/kg)	0.104 ± 0.003	0.11 ± 0.002	283	.119
Mn (g/kg)	0.90 ± 0.06	0.91 ± 0.04	226	.900

*Note*: Bold values indicate statistically significant.

Abbreviations: SMM, soil moisture; SOC, soil organic carbon.

*p < .05; **p < .01; ***p < .001.

## 3 RESULTS

### 3.1 SPCP characteristics of licks

Consistent with the bare soil status, the SOC and N at licks were lower than those of the matrix by 34.5%–36.2% (SOC: lick = 197.17 ± 10.62 g/kg, matrix = 152.34 ± 6.03 g/kg, *W* = 360, *p* < .001; N: lick = 4.69 ± 0.52 g/kg, matrix = 7.16 ± 0.31 g/kg, *W* = 351, *p* < .001. *n*_lick_ = *n*_matrix_ = 21; Table 1). Soil moisture (SMM) was significantly lower at licks than matrix (lick = 11.92 ± 1.01%, matrix = 16.51 ± 1.15%, *W* = 330, *p* = .005).

Among the 10 measured mineral contents, only Na, Fe, and Al were significantly higher at licks (Na: lick = 8.86 ± 0.37 g/kg, matrix = 7.82 ± 0.14 g/kg, *W* = 133, *p* = .028; Fe: lick = 35.86 ± 1.56 g/kg, matrix = 31.28 ± 0.92 g/kg, *W* = 138.5, *p* = .040; Al: lick = 45.22 ± 1.67 g/kg, matrix = 40.83 ± 0.57 g/kg, *W* = 86, *p* < .001; Table 1).

### 3.2 Deer visitation pattern and the “sodium supplementation” hypothesis

In total, 4882 videos of sika deer activity were captured using 21 camera traps over the four‐month investigation, with 1421 independent events. Data from six cameras were removed from the following calculations because of missing records for 1–2 months. The intensity of deer visitation varied greatly among licks (RAI = 63.9 ± 40.0, *n* = 15).

**FIGURE 3:**
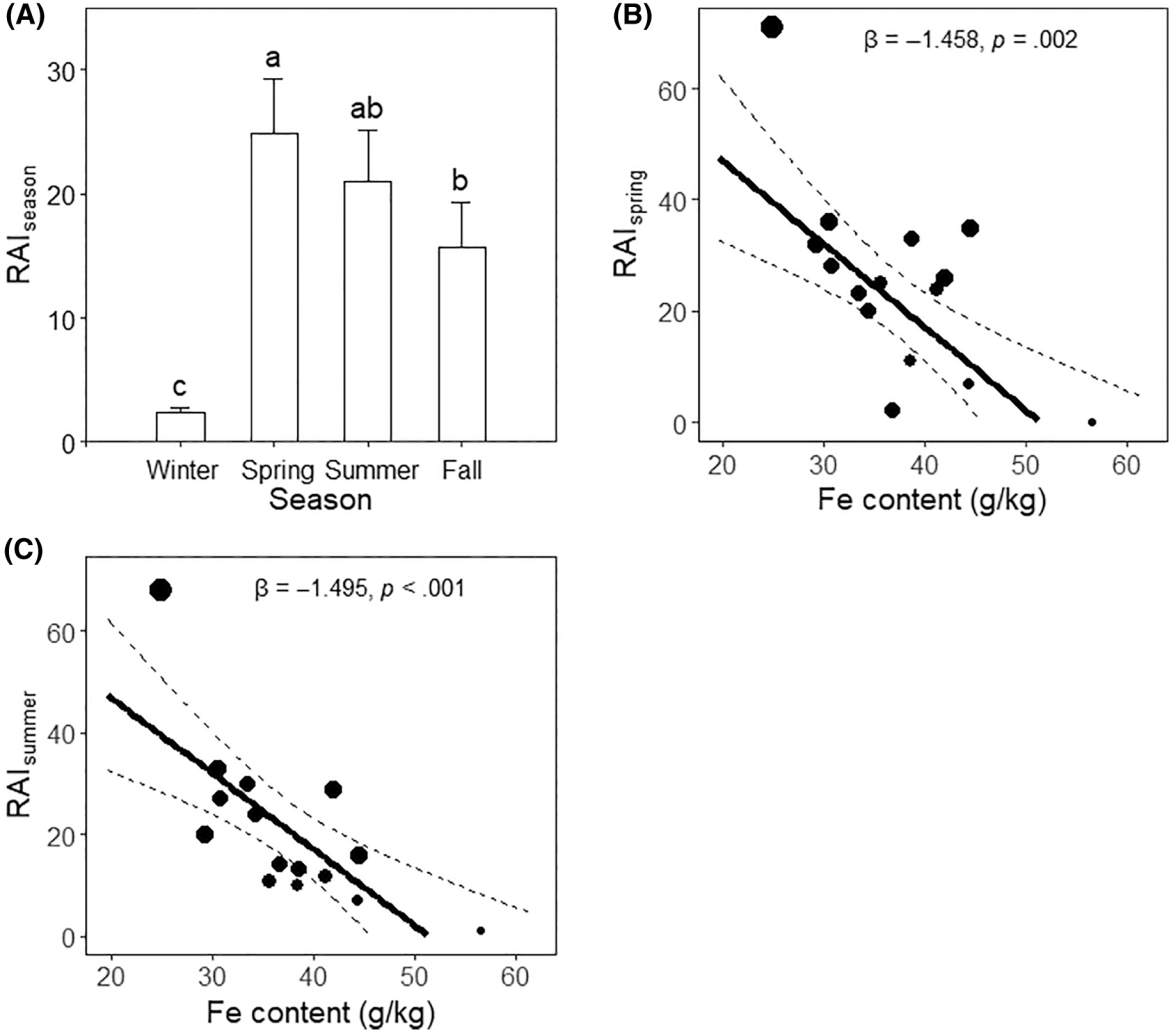
Deer visitation pattern captured by camera trap set near licks. (A) Seasonal variation of deer visitation intensity. Kruskal–Wallis rank sum test was applied to compare the intensity differences between seasons, the result of which was shown by the lowercase letter. (B) and (C) shows the weighted linear regression models in explaining the spatial pattern of deer visitation during spring and summer. The size of the points represents the weight of each lick. Larger points indicate higher overall deer visitation intensity (RAI) for that lick and greater relative importance in the regression model.

Among the four seasons, spring had the highest intensity of deer visitation (${\mathrm{RAI}}_{\text{spring}}$ = 24.867 ± 4.453), significantly higher than that of winter (${\mathrm{RAI}}_{\text{winter}}$ = 2.333 ± 0.475, *p* < .001) and fall (${\mathrm{RAI}}_{\text{fall}}$ = 15.667 ± 3.630, *p* = .026), but only marginally higher than that of summer (${\mathrm{RAI}}_{\text{summer}}$ = 21 ± 4.129, *p* = .068). The intensity during the summer was marginally higher than that during the fall (*p* = .061). The winter season had the lowest visitation intensity (*p* < .001) (Figure 3A).

We performed the weighted linear regression models between deer visitation and mineral contents only during the most intensive season (spring and summer). We found none significant models between sodium content and either ${\mathrm{RAI}}_{\text{spring}}$ (*p* = .373) or ${\mathrm{RAI}}_{\text{summer}}$ (*p* = .241) (Table S3). All minerals except Fe had no significant pattern with deer visitation (*p* > .105). In both spring and summer, deer visitation showed an avoidance of high Fe content at licks (spring: β = −1.458, *p* = .002, Figure 3B, summer: β = −1.495, *p* < .001, Figure 3C and Table S3).

### 3.3 The profile of soil microbes

In total, 8480 bacteria OTUs and 6890 fungal OTUs at ≥97% similarity were identified from the 42 soil samples. The bacterial OTUs belonged to 45 phyla and 944 genera, whereas the fungal OTUs belonged to 16 phyla and 992 genera. All 45 phyla of bacteria were found in both the licks and the matrix, and the most common phyla were *Probeobacteria* and *Actinobacteriota*. All 16 phyla of fungi were found in both the licks and the matrix, and the most common phylum was *Ascomycota*.

#### 3.3.1 Bacteria

**FIGURE 4:**
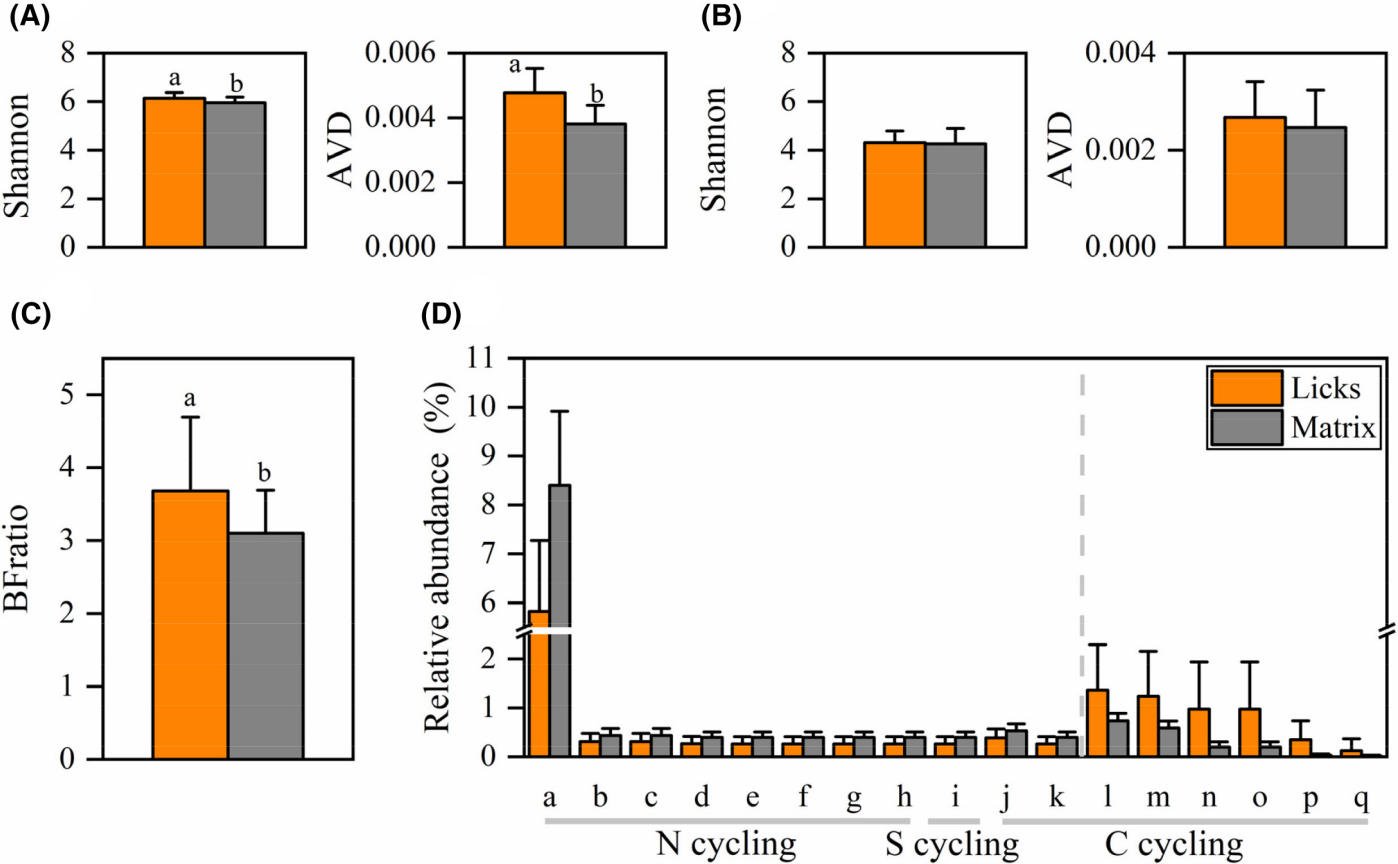
Soil microbes' differences between licks and matrix. (A) Diversity (Shannon) and variation (AVD) differences of bacteria community. (B) Shannon and AVD differences of fungal community. (C) Difference in relative abundance ratio of bacteria to fungi (BFratio). (D) Difference in relative abundances of predicted bacteria functions by FAPROTAX. Only functions with significant abundance differences between licks and matrix were shown. For functions in the left of the broken gray line, the abundances are significantly higher at licks than that at the matrix. The situation is opposite on the right of the broken line. a: Nitrogen fixation; b: Nitrate respiration; c: Nitrogen respiration; d: Nitrite respiration; e: Nitrite denitrification; f: Nitrous oxide denitrification; g: Nitrate denitrification; h: Denitrification; i: Anoxygenic photoautotrophy S oxidizing; j: Photoheterotrophy; k: Anoxygenic photoautotrophy; l: Phototrophy; m: Photoautotrophy; n: Cyanobacteria; o: Oxygenic photoautotrophy; p: Chloroplasts; q: Methanol oxidation; The left part of the dotted line indicates that the values at the licks are significantly higher than the matrix, and the right part has the opposite trend.

Community diversity indicated by the Shannon index was significantly higher at licks than at the matrix (licks: 6.14 ± 0.05, matrix: 5.96 ± 0.05. *W* = 138, *p* = .038. Figure 4A). Communities at licks were more variant than those at the matrix (AVD: licks: 0.0048 ± 0.000, matrix: 0.0038 ± 0.000. *W* = 66, *p* < .001, Figure 4A).

From phylum to OTUs, 82 clades were more abundant in the licks, and 61 clades were more abundant at the matrix (Figures S2 and S3). Four phyla (*Chloroflexi*, *Gemmatimonadota*, *Cyanobacteria*, and *Patescibacteria*) significantly increased at the licks, whereas three phyla (*Proteobacteria*, *Verrucomicrobiota*, and *Planctomycetota*) significantly increased at the matrix (Figure S1a).

**FIGURE 5:**
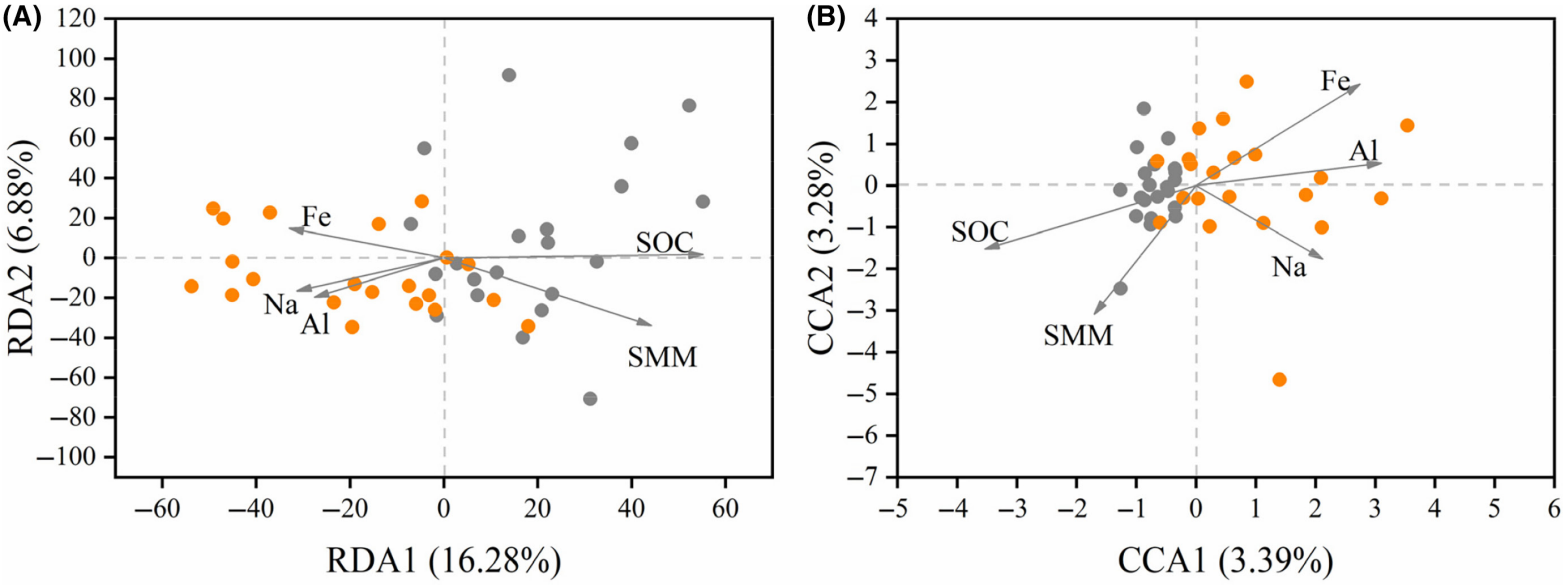
The relationship between microbial communities and soil physicochemical properties (SPCP). (A) Redundancy analysis (RDA) result shows the bacterial communities and (B) canonical correspondence analysis (CCA) result shows fungal communities.

Among the five SPCPs tested, only SOC, SMM, and Fe were significantly associated with the first two axes of RDA analysis. The two axes could explain 23.16% of the total variance in bacterial communities, and the community composition of the licks and matrix was mainly differentiated along the first axis. SOC had the strongest association, which was much stronger on the first axis than on the second (Figure 5A and Table S4). These patterns suggest that SOC had the most important effect on the differentiated bacterial communities at licks.

In total, 1361 bacteria OTUs were assigned to 58 functional groups. The relative abundances of the six functional groups significantly increased at the licks. All increased functions of licks were related to carbon cycling in ecosystems, 83.3% (5/6) of which were carbon fixation functions through photosynthesis processes. Relative abundances of 11 functional groups were significantly increased at the matrix, 72.7% (8/11) of which were related to nitrogen cycling (Figure 4D).

#### 3.3.2 Fungi

Both the Shannon and AVD indices of fungal communities were not significantly different between licks and matrix (Shannon: licks: 4.31 ± 0.10, matrix: 4.26 ± 0.14, *W* = 227, *p* = .881; AVD: licks: 0.0027 ± 0.0002, matrix: 0.0025 ± 0.0002, *W* = 189, *p* = .440. Figure 4B).

From phylum to OTU, 94 clades were more abundant at licks, and 71 clades were more abundant at the matrix (Figures S4 and S5). *Ascomycota* was the most abundant phylum at licks, whereas *Mortierellomycota* and *Rozellomycota* were the most abundant phyla at the matrix (Figure S1b).

All five tested SPCP indices were significantly associated with the first two axes of CCA analysis. The two axes could explain 6.67% of the total variance of the fungal communities and the community composition of licks and matrix was mainly differentiated along the first axis. SOC had the strongest association, which was stronger on the first axis than on the second (Figure 5B and Table S4). These patterns suggest that SOC was also the most important factor in determining differentiated fungal communities at licks.

In total, 46 OTUs significantly differed between the licks and the matrix, of which only 20 could confidently refer to the nine functional groups (Table S5). For two functional groups, i.e., dung saprotroph and fungal parasite, two OTUs were more abundant at the licks, whereas for the other two functional groups, i.e., animal pathogen and ectomycorrhizal, three OTUs were more abundant at the matrix. For the remaining five functional groups, each contained one OTU that was more abundant at the matrix, while 1–7 OTUs were more abundant at the licks.

#### 3.3.3 BFratio

The BFratio was significantly higher at licks (licks: 3.68 ± 0.22, matrix: 3.10 ± 0.13, *W* = 132, *p* = .026) (Figure 4C).

### 3.4 The effect of deer visitation on soil microbial community structure

**FIGURE 6:**
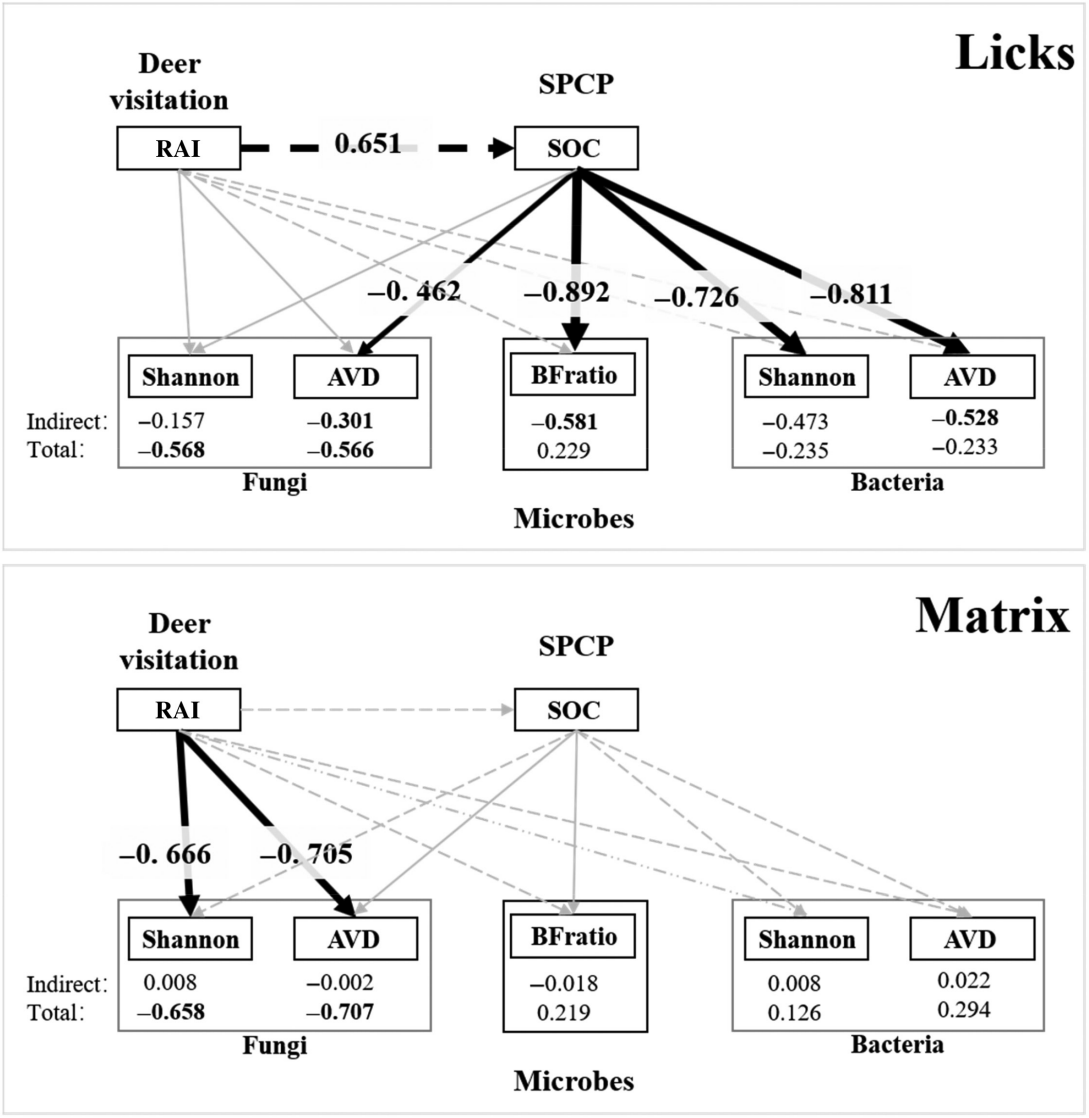
Effects of deer visitation on soil microbial community structure at licks and matrix based on partial least square structural equation model (PLS‐SEM). Multigroup analysis (MGA) suggests licks and matrix have divergent effects of deer visitation. Dashed line and solid line represent positive and negative standardized path coefficients, respectively. Black and gray line represents significant (*p* ≤ .05) and non‐significant (*p* > .05) standardized path coefficients, respectively. Widths of significant paths are scaled based on standardized path coefficients. More detailed results can be found in Table 2.

The complete PLS‐SEM results for the five microbial indices are presented in Table 2 and Figure 6. The results presented below were the standardized path coefficient estimation (denoted by “$\beta_{\text{path}\left(\text{effect type}\right)}$,” with “path” name and “effect type” identical to those listed in Table 2. Subscript “effect type” was listed only when the path “RAI→microbes” could not differentiate between the direct effect and the total effect of RAI on microbes).

**TABLE 2:** Partial least square structural equation model (PLS‐SEM) result shows the effect of deer visitation on soil microbial community structure at licks and matrix.

Microbes	Indices of microbial community structure	Effect types	Path	Licks			Matrix			Bootstrap MGA (licks vs. matrix)	
				Path coefficient (β)	Confidence intervals	*p* value	Path coefficient (β)	Confidence intervals	*p* value	Difference	*p* value
Fungi	Shannon	Direct	RAI→Shannon	−.411	[−0.895, 0.334]	.243	**−.666**	**[−1.005, 0.026]**	**.017**	0.256	.566
			RAI→SOC	**.651**	**[0.324, 0.822]**	**.000**	.077	[−0.522 0.641]	.805	0.574	.078
			SOC→Shannon	−.241	[−0.705, 0.228]	.344	.106	[−0.389, 0.504]	.630	−0.347	.283
		Indirect	RAI→SOC→Shannon	−.157	[−0.499, 0.132]	.383	.008	[−0.181, 0.245]	.938	−0.165	.390
		Total	RAI→Shannon	**−.568**	**[−0.906, 0.051]**	**.035**	**−.658**	**[−0.929, −0.115]**	**.006**	0.090	.800
	AVD	Direct	RAI→AVD	−.265	[−0.669, 0.245]	.271	**−.705**	**[−0.956, −0.237]**	**.000**	0.440	.131
			RAI→SOC	**.651**	**[0.324, 0.822]**	**.000**	.077	[−0.522 0.641]	.805	0.574	.078
			SOC→AVD	**−.462**	**[−0.788, −0.029]**	**.015**	−.026	[−0.513, 0.480]	.922	−0.436	.178
		Indirect	RAI→SOC→AVD	**−.301**	**[−0.564, −0.026]**	**.035**	−.002	[−0.247, 0.155]	.983	−0.299	.072
		Total	RAI→AVD	**−.566**	**[−0.889, −0.029]**	**.013**	**−.707**	**[−0.904, −0.351]**	**.000**	0.141	.593
Bacteria	Shannon	Direct	RAI→Shannon	.238	[−0.432, 0.792]	.426	.118	[−0.564, 0.668]	.712	0.120	.798
			RAI→SOC	**.651**	**[0.324, 0.822]**	**.000**	.077	[−0.522 0.641]	.805	0.574	.078
			SOC→Shannon	**−.726**	**[−1.294, −0.074]**	**.023**	.106	[−0.265, 0.539]	.590	**−0.832**	**.031**
		Indirect	RAI→SOC→Shannon	−.473	[−1.120, −0.080]	.072	.008	[−0.096, 0.374]	.932	**−0.481**	**.047**
		Total	RAI→Shannon	−.235	[−0.582, 0.252]	.244	.126	[−0.444, 0.615]	.654	−0.361	.310
	AVD	Direct	RAI→AVD	.295	[−0.409, 0.996]	.397	.272	[−0.459, 0.726]	.373	0.023	.985
			RAI→SOC	**.651**	**[0.324, 0.822]**	**.000**	.077	[−0.522 0.641]	.805	0.574	.078
			SOC→AVD	**−.811**	**[−1.359, −0.179]**	**.006**	.285	[−0.195, 0.621]	.176	**−1.096**	**.007**
		Indirect	RAI→SOC→AVD	**−.528**	**[−1.174, −0.140]**	**.042**	.022	[−0.140, 0.418]	.853	**−0.550**	**.030**
		Total	RAI→AVD	−.233	[−0.640, 0.393]	.330	.294	[−0.389, 0.731]	.323	−0.540	.190
Bacteria and Fungi	BFratio	Direct	RAI→BFratio	.810	[−0.222, 1.370]	.051	.237	[−0.204, 0.706]	.280	0.573	.248
			RAI→SOC	**.651**	**[0.324, 0.822]**	**.000**	.077	[−0.522 0.641]	.805	0.574	.078
			SOC→BFratio	**−.892**	**[−1.266, −0.539]**	**.000**	−.236	[−0.628, 0.269]	.283	**−0.657**	**.022**
		Indirect	RAI→SOC→BFratio	**−.581**	**[−0.987, −0.272]**	**.001**	−.018	[−0.328, 0.150]	.875	**−0.563**	**.010**
		Total	RAI→BFratio	.229	[−0.598, 0.850]	.558	.219	[−0.230, 0.589]	.278	0.010	.949

*Note*: Bold values indicate statistically significant.

#### 3.4.1 Bacteria

Deer visitation had similar effects on the two indices of bacteria (AVD, Shannon) whether in the licks or the matrix.

At licks, RAI had an indirect negative effect on both Shannon and AVD via SOC (Shannon: $\beta_{\mathrm{RAI}\to \mathrm{SOC}\to \text{Shannon}}=-0.473$, *p* = .072; AVD: $\beta_{\mathrm{RAI}\to \mathrm{SOC}\to \mathrm{AVD}}=-0.528$, *p* = .042). Increased deer visitation elevated SOC ($\beta_{\mathrm{RAI}\to \mathrm{SOC}}=0.651$, *p* = .000), which then reduced the bacteria diversity and increased community stability (Shannon: $\beta_{\mathrm{SOC}\to \text{Shannon}}=-0.726$, *p* = .023; AVD: $\beta_{\mathrm{SOC}\to \mathrm{AVD}}=-0.811$, *p* = .006). In contrast to the indirect effects, the direct effects of RAI were weaker and insignificant (Shannon: $\beta_{\mathrm{RAI}\to \text{Shannon}\left(\text{direct}\right)}=0.238$, *p* = .426; AVD: $\beta_{\mathrm{RAI}\to \mathrm{AVD}\left(\text{direct}\right)}=0.295$, *p* = .397).

At the matrix, both the direct and indirect effects of RAI were insignificant. The direct effects of RAI were comparable to licks (bootstrap MGA: *p* > .7), while the indirect effects of RAI were much weaker than that at licks (Shannon: $\beta_{\mathrm{RAI}\to \mathrm{SOC}\to \text{Shannon}}=0.008$, *p* = .932, bootstrap MGA: *p* = .047; AVD: $\beta_{\mathrm{RAI}\to \mathrm{SOC}\to \mathrm{AVD}}=0.022$, *p* = .853, bootstrap MGA: *p* = .030). The extremely weaker indirect effects of RAI at the matrix were caused by the superposition of significantly weaker effects of SOC on bacterial community structure (bootstrap MGA: *p* = .031 and .007 for Shannon and AVD, respectively) and marginally weaker effects of RAI on SOC (bootstrap MGA: *p* = .078).

#### 3.4.2 Fungi

The influence of deer visitation on the fungal community structure was comparable between licks and the matrix (bootstrap MGA: *p* > .07). Deer visitation directly reduced the fungi diversity and increased community stability, with a significant path coefficient at the matrix (Shannon: $\beta_{\mathrm{RAI}\to \text{Shannon}\left(\text{direct}\right)}=-0.666$, *p* = .017; AVD: $\beta_{\mathrm{RAI}\to \mathrm{AVD}\left(\text{direct}\right)}=-0.705$, *p* = .000), while weaker and insignificant at licks (Shannon: $\beta_{\mathrm{RAI}\to \text{Shannon}\left(\text{direct}\right)}=-0.411$, *p* = .243; AVD: $\beta_{\mathrm{RAI}\to \mathrm{AVD}\left(\text{direct}\right)}=-0.265$, *p* = .271). Most indirect effects of RAI via SOC in both matrix and licks were insignificant except RAI on AVD at licks ($\beta_{\mathrm{RAI}\to \mathrm{SOC}\to \mathrm{AVD}}$ = −0.301, *p* = .035). Collectively, the total effects of RAI were concordant with direct effects, while all total effects were significant for both indices and in both licks and matrix (licks: Shannon: $\beta_{\mathrm{RAI}\to \text{Shannon}\left(\text{total}\right)}=-0.568$, *p* = .035; AVD: $\beta_{\mathrm{RAI}\to \mathrm{AVD}\left(\text{total}\right)}=-0.566$, *p* = .013; matrix: Shannon: $\beta_{\mathrm{RAI}\to \text{Shannon}\left(\text{total}\right)}=-0.658$, *p* = .006; AVD: $\beta_{\mathrm{RAI}\to \mathrm{AVD}\left(\text{total}\right)}=-0.707$, *p* = .000).

#### 3.4.3 BFratio

The pattern of deer visitation influencing the BFratio was similar to the Shannon and AVD patterns of bacteria. At licks, RAI had an indirect negative effect on the BFratio via SOC, with intensified deer visitation and a lower relative abundance of bacteria ($\beta_{\mathrm{RAI}\to \mathrm{SOC}\to \text{BFratio}}=-0.581$, *p* = .001). Unlike bacteria, the direct effect of RAI was larger and positive, yet only marginally significant ($\beta_{\mathrm{RAI}\to \text{BFratio}\left(\text{direct}\right)}=0.810$, *p* = .051).

Similar to bacteria, both the direct and indirect effects of RAI on the BFratio were insignificant at the matrix. The indirect effect of RAI on BFratio at the matrix was much weaker than at the licks ($\beta_{\mathrm{RAI}\to \mathrm{SOC}\to \text{BFratio}}=-0.018$, *p* = .875, bootstrap MGA: *p* = .010) due to superposition between significantly weaker effects of SOC on BFratio (bootstrap MGA: *p* = .022), and marginally weaker effects of RAI on SOC (bootstrap MGA: *p* = .078).

## 4 DISCUSSION

Our study revealed that licks are small islands in the firebreak with specific SPCP and soil microbes that diverge from the surrounding matrix. Carbon and nitrogen contents were the most important factors in determining the unique composition and structure of microbes at licks. Deer visitations during the growing season were more intense than those in other seasons, and deer exhibited strong avoidance patterns at licks with high iron. Their visitations divergently influenced the carbon and nitrogen inputs between the licks and the matrix, which may indirectly influence the microbial stability of the licks.

### 4.1 The geophagy function at licks is mainly sodium supplementation for sika deer during the growing season

As expected due to the “sodium supplementation” hypothesis, sodium content was significantly higher at licks than at surrounding matrix, though a large variation existed among licks. Of the 10 measured minerals, iron and aluminum were the two other minerals that were significantly higher at licks. Nonetheless, we argue that two results from this study support sodium as the main mineral at licks supplement for deer.

First, although only data of four representative months was used, the intensity of visitation to licks clearly increased during the growing season. The growing season is considered to be a period of higher sodium demand for sika deer due to the lactation stage requiring additional sodium absorption, as well as a deficiency of sodium content in food plants during this period (Atwood & Weeks, 2002; Pletscher, 1987; Tracy & McNaughton, 1995). Nonetheless, in addition to the demand for geophagy, intensified deer visitations during the growing season can be generated by more abundant food during this period (Humphries et al., 2017). According to a diet study and vegetation investigation, sika deer only consume 7%–14% herbaceous food during growing season in the firebreak. Compared to the forest habitat, the grassland in firebreak is not an optimal food habitat for sika deer (Wen P, Zhu D, Wang L, Wu F, Bao L, Wang T, Ge J and Wang H, under review). Hence, abundant food during growing season is not a main reason for the intensified deer visitations. Additionally, artificial salt sprayed in front of cameras during spring and summer is used to increase detection rate of sika deer in the National Park (another experiment conducted by T.W.), which can support the sodium‐supplementation demand for sika deer.

Second, iron and aluminum are unlikely to be the main supplementing minerals. To our knowledge, none existing studies have reported the deficiency of iron and aluminum in diets of ungulates. While iron is vital for almost all living organisms, iron deficiency is rare for ruminants who start eating plants, rather than consuming breast milk, early (Berger, 2006). Iron overdose is toxic. It can cause various diseases and problems, such as liver damage, dehydration, low blood pressure, and nervous disorders (McDonald et al., 2010). Hence, iron avoidance visitation patterns may be a strategy helping limit the risk of iron overdose. Aluminum is also a toxin for LMH (Crowe et al., 1990; Kumar & Gill, 2009). Toxicity symptoms include decreased feed intake, reduced efficiency in converting feed to body weight gain, hypercalcemia, reduced bone mineralization, and accumulation of aluminum in the body tissues (Crowe et al., 1990; Fontenot et al., 1989). Aluminum can also reduce phosphate and magnesium absorption, which can result in uncoordinated gait, convulsions, coma, or even death due to low magnesium levels in the blood (“grass tetany” (Allen et al., 1990; Fontenot et al., 1989; Martens & Schweigel, 2000)). Our cameras occasionally recorded grass tetany‐like behavior at licks only (Video S1), which may implicate the potential toxicity of aluminum for deer.

Nonetheless, the intensity of deer visitation has no discernible signal with sodium content at licks. Four reasons may explain the absence. First, attraction by mineral licks is only one of the many factors that influence the deer visitation pattern. Other factors, such as the quality of food habitat, risk landscape heterogeneity generated by predators, can largely influence the spatial distribution of deer population and then the visitation events recorded by camera traps (Emerson et al., 2011; Miranda et al., 2010; Moser et al., 2006; Riginos & Grace, 2008). Second, the habitat quality of mineral licks determined by sodium content may be nonlinear, with a potential threshold altered the attraction pattern caused by sodium. However, little knowledge is known about the amount of sodium deficiency for ungulates in this region. Third, additional variance may come from the season definition. The seasons defined here are arbitrary, which may not completely overlap with the physiological change of sika deer. A full‐year dataset of deer visitation and a better time division may solve this problem. Finally, our sample size is small, only 15 cameras with no missing data. Considering the complex factors probably involved in the deer visitation pattern, the small sample size may have limited power to distinguish the effects of sodium attraction from the complex background. In fact, the sample size in this study is larger more than many existing studies, due to the difficulties in finding mineral licks (Griffiths et al., 2023). More efforts are required in future to increase sample size to test the utilizing pattern driven by characteristics of mineral licks.

Although the mineral distribution and deer visitation pattern conform to the “sodium supplementation” hypothesis, both of which are the common way to test geophagy function (Clayton & MacDonald, 1999; Lavelle et al., 2014; Monaco et al., 2019; Owen et al., 2014), they are all indirect evidence and uncertainties still exist. Other than “sodium supplementation,” our study cannot exclude other geophagy functions at licks. Although most measured minerals have comparable contents at licks as the surrounding matrix, LMH can still benefit from ingesting co‐occurring minerals at licks (Ayotte et al., 2006; Molina et al., 2014). Hence, more direct evidence of the geophagy function is needed in the future. For example, data on sodium and other minerals in food plants and LMH itself should be collected to evaluate the extent of deficiency of these minerals across seasons. The exact risk of mineral deficiency or mineral overdose in animal models of LMH species themselves should also be evaluated. Cafeteria‐type experiments can be designed to distinguish the effects of minerals during geophagy.

### 4.2 Soil microbial community at licks: altered biogeochemical cycles and increased vulnerability

To the best of our knowledge, our study is the first to characterize the microbial profile of licks. Our study shows that licks sustain divergent microbes from the surrounding matrix, with carbon and nitrogen content being the most important determinants of both bacterial and fungal communities. Abundant bacteria (such as *Chloroflexi*, *Gemmatimonadota, Cyanobacteria*, and *Patescibacteria*) and fungi (such as *Ascomycota*) found at licks are usually pioneers in nutrient‐poor soils or bare rocks (Kumar et al., 2022; Liu et al., 2019). As the composition of microbes changes, the low availability of carbon and nitrogen at licks also alters biogeochemical cycles. For example, nitrogen fixation is largely reduced at licks, which lowers the nitrogen input and suppresses other processes in nitrogen cycling. For carbon cycling, many photoautotrophs increase their abundance at licks, such as *Chloroflexi* and *Cyanobacteria*, the bacteria of which have been reported to be able to utilize CO_2_ and perform photosynthesis (Burganskaya et al., 2018; Burow et al., 2013). In contrast, bacteria that utilize organic carbon (photoheterotrophy) are less abundant at licks because of their low carbon content. Therefore, the microbes at licks and their corresponding biogeochemical cycles are mainly driven by the low carbon and nitrogen contents.

Another major feature of microbes at licks is their instability, which is supported by the following findings. First, bacteria showed more pronounced changes than fungi (Shannon and BFratio) when comparing licks and the matrix. The more significant bacterial pattern was consistent with the expectation of community changes under harsh and frequently disturbed environments (de Vries et al., 2018; Xun et al., 2018). Second, the bacterial communities were more variable at the licks. The larger variance among communities implies that random processes increase their role in shaping the bacterial communities at licks. The microbial community structure implies that the licks are unstable and probably under frequent disturbance, the state of which concurs with the disturbance from deer visitation. Finally, according to PLS‐SEM, the microbial community at the licks was more sensitive to soil carbon and nitrogen changes than that at the matrix. The vulnerability of a system describes its degree of susceptibility and restoration ability to perturbations (Weißhuhn et al., 2018). The unstable state and susceptibility to SPCP changes suggest the vulnerability of licks to environmental changes. For example, climate warming can increase plant growth and carbon inputs (Jansson & Hofmockel, 2020), which can drive unstable licks to an alternative stable state and increase the risk of their disappearance. In addition, vulnerability also makes licks susceptible to LMH density changes caused by natural or human disturbances.

### 4.3 The role of deer in maintaining the stability of soil microbial community at licks

Although the LMH are generally considered ecosystem engineers at licks (Ghanem & Voigt, 2014; Weir, 1969), how the LMH influence and maintain licks has rarely been studied. Our path analysis clearly shows a much stronger indirect effect of deer on soil microbes at licks than at the matrix, especially for bacteria, which provide clues for understanding the ecosystem engineering role of the LMH. Intensified deer visitation can indirectly promote the stability of microbial community (lower community variability) by altering carbon and nitrogen inputs. Several deer behaviors at licks may promote the stabilizing processes. First, the bare soil status, the low carbon, and nitrogen at licks determine the microbial community composition and sensitivity of community stability to deer visitation. Frequent herbivory and trampling at licks can maintain the bare soil status. Sika deer can quickly remove seedlings at licks, according to our camera trap data (Video S3). These disturbances determine the instability of soil microbes at licks. Second, compared to grazing and trampling, which result in lower carbon and nitrogen inputs, excretion is a major way to elevate carbon and nitrogen inputs. Owing to the bare soil status of licks, excretion is probably the primary carbon and nitrogen input, which is supported by the stronger positive effect of deer visitation on the carbon content at licks than at the matrix. The community stability of both bacteria and fungi at licks can benefit from increased deer visitation, which brings more carbon and nitrogen inputs due to excretion. Hence, grazing, trampling, and excretion are all involved in shaping the microbes at licks, whereas excretion is the main behavior stabilizing the microbial community at licks.

Because our study is the first to characterize soil microbes and show the processes by which LMH maintain microbes at licks, it is unknown how the general instability features of soil microbes and the stabilizing roles of LMH at licks are revealed here. We argue that the pattern revealed in this study is not unique. For example, African elephants have been reported to create and maintain licks in depressions in savanna ecosystems (Weir, 1969). Hunting LMH resulted in the disappearance of licks from tropical forest ecosystems in western Amazonia (Ghanem & Voigt, 2014). However, such examples are rare, and importantly, they only provide indirect evidence for the role of the LMH. Licks are vital for both large and small herbivores. However, further studies involving more herbivore species and more types of licks are required before more general conclusions can be reached.

## 5 CONCLUSION

Our study provides ample evidence for a close relationship between licks and large herbivores. The geophagy of sika deer at licks mainly involves supplementation with sodium during the growing season. Licks are vulnerable due to their unstable soil microbe communities and susceptibility to carbon and nitrogen perturbation. Sika deer play critical roles in shaping and stabilizing microbe communities at licks through behaviors such as grazing, trampling, and excretion. Our results suggest that the conservation of natural licks relies on their continued use by large mammalian herbivores like sika deer.

## AUTHOR CONTRIBUTIONS

**Peiying Wen:** Conceptualization (supporting); data curation (lead); formal analysis (lead); investigation (lead); methodology (lead); visualization (lead); writing – original draft (lead); writing – review and editing (equal). **Feng Wu:** Investigation (supporting); writing – review and editing (supporting). **Lei Bao:** Project administration (lead); resources (lead). **Tianming Wang:** Funding acquisition (supporting); project administration (equal); resources (equal). **Jianping Ge:** Funding acquisition (lead); resources (lead). **Hongfang Wang:** Conceptualization (lead); formal analysis (equal); funding acquisition (lead); methodology (equal); visualization (supporting); writing – original draft (supporting); writing – review and editing (lead).

## CONFLICT OF INTEREST STATEMENT

The authors declare that they have no competing financial interests or personal relationships that may have influenced the work reported in this study.

## Supporting information


Data S1:

Video S1

Video S2

Video S3

## Data Availability

The data for our study are openly available in the NCBI at https://www.ncbi.nlm.nih.gov/sra/ PRJNA764365 accessed on 18 September 2021, reference number PRJNA764365.
