# Supplementary material for: The role of large mammalian herbivores in shaping and maintaining soil microbial communities of natural mineral licks: A case study on sika deer at the firebreak adjacent to the Sino‐Russian border
Source: Ecol Evol. 2024 Feb 1;14(2):e10878. doi: 10.1002/ece3.10878 (PMC10834104; doi:10.1002/ece3.10878)
Supplement: Supplementary file 1 — Data S1: [file ECE3-14-e10878-s001.docx]

**Supplementary Material and Appendices**

Table S1 Acronym definition and meaning

| Acronyms | Meaning |
| --- | --- |
| licks | Natural mineral licks |
| matrix | The background area within a firebreak, which is distinct from the licks and forms the overall environment of the firebreak |
| LMH | Large mammal herbivores |
| SPCP | Soil physicochemical properties |
| SOC | Soil organic carbon |
| N | Soil total nitrogen |
| SMM | Soil moisture |
| Na | Sodium |
| Fe | Iron |
| Al | Aluminum |
| P | Phosphorus |
| K | Potassium |
| Ca | Calcium |
| Mg | Magnesium |
| Cu | Copper |
| Zn | Zinc |
| Mn | Manganese |
| RAI_season_ | The number of independent deer visitation events was determined for each sampling month |
| RAI | The overall deer visitation intensity of each lick |
| RAI_spring_ | The number of independent deer visitation events was determined for May |
| RAI_summer_ | The number of independent deer visitation events was determined for Autumn |
| OTUs | Operational taxonomic units |
| Shannon | The Shannon-Wiener index |
| AVD | The average variation degree of the microbial community |
| BFratio | The relative abundance ratio of bacteria to fungi |
| ANOSIM | Analysis of similarity |
| RDA | Redundancy analysis |
| CCA | Canonical correspondence analysis |
| SEM | Structural equation model |
| PLS-SEM | Partial least squares structural equation model |
| bootstrap MGA | Bootstrap multigroup analysis |

Table S2 Spearman correlations between soil physicochemical properties (SPCP).

|  | SOC | N | SMM | Na | Fe | Al | P | K | Ca | Mg | Cu | Zn | Mn |
| --- | --- | --- | --- | --- | --- | --- | --- | --- | --- | --- | --- | --- | --- |
| SOC |  | 0.84** | 0.53* | -0.47* | -0.73** | -0.16 | 0.58** | -0.19 | 0.31 | -0.10 | 0.53* | 0.44* | 0.63** |
| N | 0.96** |  | 0.77** | -0.68** | -0.61** | -0.06 | 0.73** | -0.34 | 0.51* | 0.20 | 0.75** | 0.64** | 0.60** |
| SMM | 0.32 | 0.30 |  | -0.48* | -0.51* | -0.04 | 0.79** | -0.27 | 0.45* | 0.12 | 0.64** | 0.53* | 0.62** |
| Na | -0.18 | -0.16 | 0.41 |  | 0.19 | 0.03 | -0.42 | 0.18 | -0.37 | -0.54* | -0.58** | -0.62** | -0.42 |
| Fe | -0.60** | -0.54* | -0.36 | -0.33 |  | 0.34 | -0.64** | -0.05 | -0.02 | 0.50* | -0.22 | -0.45* | -0.70** |
| Al | -0.53* | -0.49* | -0.25 | -0.21 | 0.74** |  | 0.13 | -0.32 | 0.54* | 0.28 | -0.15 | -0.11 | -0.27 |
| P | 0.76** | 0.83** | 0.37 | -0.11 | -0.28 | -0.26 |  | -0.30 | 0.51* | 0.07 | 0.39 | 0.54* | 0.55** |
| K | -0.14 | -0.24 | -0.06 | 0.29 | -0.47* | -0.444* | -0.39 |  | -0.74** | -0.55** | -0.14 | 0.19 | -0.01 |
| Ca | 0.40 | 0.46* | 0.16 | -0.40 | 0.29 | 0.34 | 0.56** | -0.80** |  | 0.61** | 0.30 | 0.15 | 0.21 |
| Mg | 0.04 | 0.10 | -0.17 | -0.67** | 0.71** | 0.499* | 0.26 | -0.76** | 0.72** |  | 0.30 | 0.11 | -0.08 |
| Cu | 0.37 | 0.41 | -0.29 | -0.76** | 0.14 | 0.13 | 0.34 | -0.39 | 0.48* | 0.49* |  | 0.56** | 0.42 |
| Zn | 0.73** | 0.73** | -0.14 | -0.49* | -0.32 | -0.494* | 0.58** | -0.06 | 0.24 | 0.17 | 0.54* |  | 0.59** |
| Mn | 0.54* | 0.61** | 0.30 | -0.32 | 0.05 | 0.20 | 0.66** | -0.69** | 0.90** | 0.53* | 0.49* | 0.31 |  |

Notes: Above the diagonal are the results of the matrix and below are the results of the licks. ***, *p* < 0.001; **,0.001 $\leq$ *p* <0.01; *, 0.01 $\leq$ *p* < 0.05;

Table S3 The result of the weighted linear regression models between deer visitation and mineral contents.

|  | *RAI_spring_* | *RAI_summer_* |
| --- | --- | --- |
| Na | *p* = 0.3729 | *p* = 0.2408 |
| P | *p* = 0.4101 | *p* = 0.1953 |
| K | *p* = 0.1546 | *p* = 0.2178 |
| Ca | *p* = 0.6454 | *p* = 0.5511 |
| Mg | *p* = 0.1684 | *p* = 0.1046 |
| Cu | *p* = 0.9855 | *p* = 0.7946 |
| Zn | *p* = 0.9544 | *p* = 0.5573 |
| Fe | ***p* = 0.0018** | ***p* = 0.0002** |
| Mn | *p* = 0.2784 | *p* = 0.1801 |
| Al | *p* = 0.2493 | *p* = 0.1293 |

Table S4 The relationship between soil physicochemical properties (SPCP) and microbial composition.

| SPCP | Bacterial communities | | | |  | Fungal communities | | | |
| --- | --- | --- | --- | --- | --- | --- | --- | --- | --- |
|  | RDA1 | RDA2 | *r^2^* | *P(>r)* |  | CCA1 | CCA2 | *r^2^* | *P(>r)* |
| SOC | 0.99 | -0.14 | 0.41 | **0.001** |  | -0.94 | -0.34 | 0.71 | **0.001** |
| Na | -0.97 | -0.25 | 0.14 | 0.056 |  | 0.80 | -0.60 | 0.37 | **0.001** |
| Fe | -0.91 | 0.42 | 0.19 | **0.020** |  | 0.79 | 0.61 | 0.62 | **0.001** |
| Al | -0.92 | -0.39 | 0.12 | 0.094 |  | 0.99 | 0.13 | 0.49 | **0.001** |
| SMM | 0.84 | -0.55 | 0.45 | **0.001** |  | -0.51 | -0.86 | 0.56 | **0.001** |

Table S5 Fungal ecosystem function predicted by FUNGuild.

|  | OTUs more abundant at matrix | OTUs more abundant at licks |
| --- | --- | --- |
| Total | 18 | 28 |
| **FUNGuild analysis summary** | | |
| Unassigned | 5 | 10 |
| Uncertain | 7 | 4 |
| Highly probable | 1 | 2 |
| Probable | 5 | 12 |
| **Function** | | |
| Dung Saprotroph |  | **1** |
| Fungal Parasite |  | **1** |
| Animal Pathogen | **2** |  |
| Ectomycorrhizal | **1** |  |
| Plant Pathogen | 1 | 7 |
| Plant Saprotroph | 1 | 1 |
| Soil Saprotroph | 1 | 1 |
| Wood Saprotroph | 1 | 2 |
| Undefined Saprotroph | 1 | 5 |





Figure S1 (a) The main phylum of bacteria that were different at the licks and matrix (LDA score > 3 and p < 0.05). (b) The main phylum of fungi that were different at the licks and matrix (LDA score > 3 and p < 0.05). All results from phylum to OTU are detailed in the Figure S2-S5.


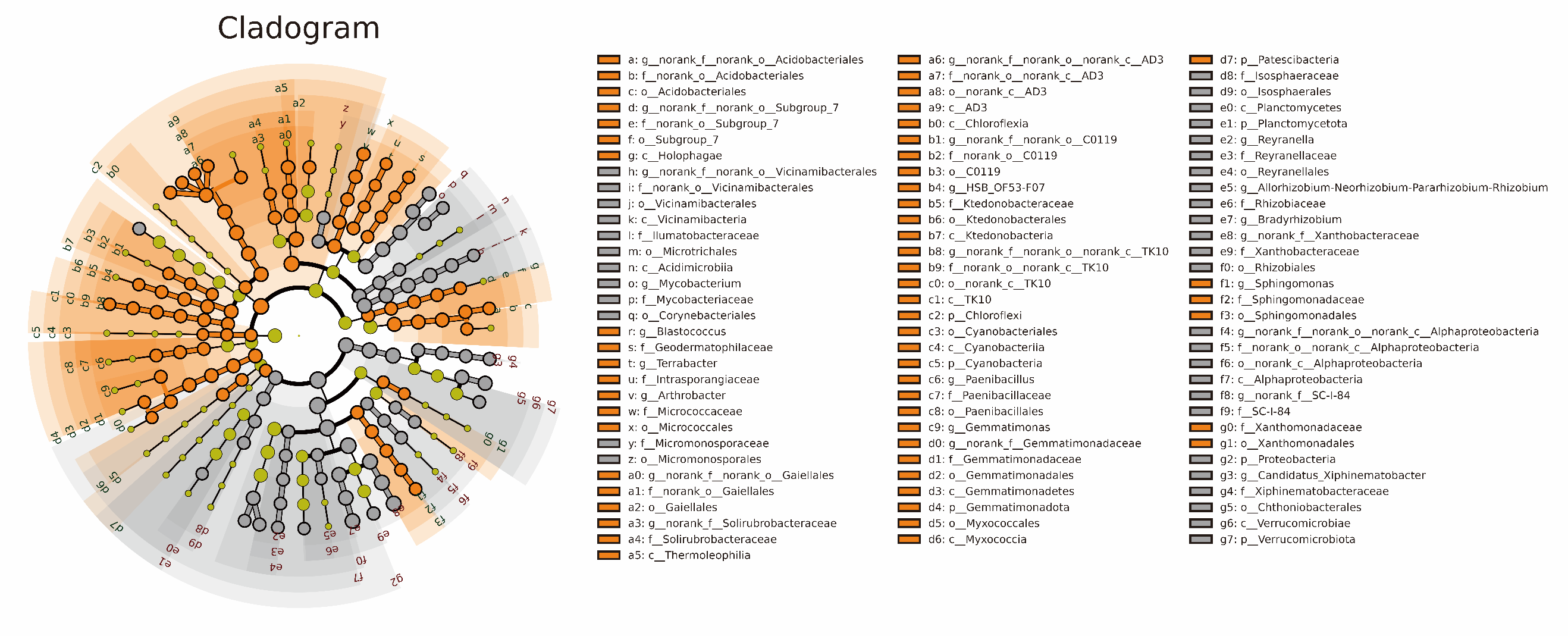


Figure S2 LEfSe analysis detected the key bacteria different between licks and the matrix. The circles from the inside to outside indicate bacteria from the kingdom to OTU levels. Yellow points represent bacteria that did not differ significantly from each other. Orange points and gray points represent bacterial biomakers of licks and the matrix, respectively. And most of the insignificant information is not shown in the figure.

Figure S3 The bar plot shows all marker bacterial species (from phylum to OTU) with LDA score greater than 3.0 (*p* < 0.05). Orange and gray represent bacterial biomakers of licks and the matrix, respectively.


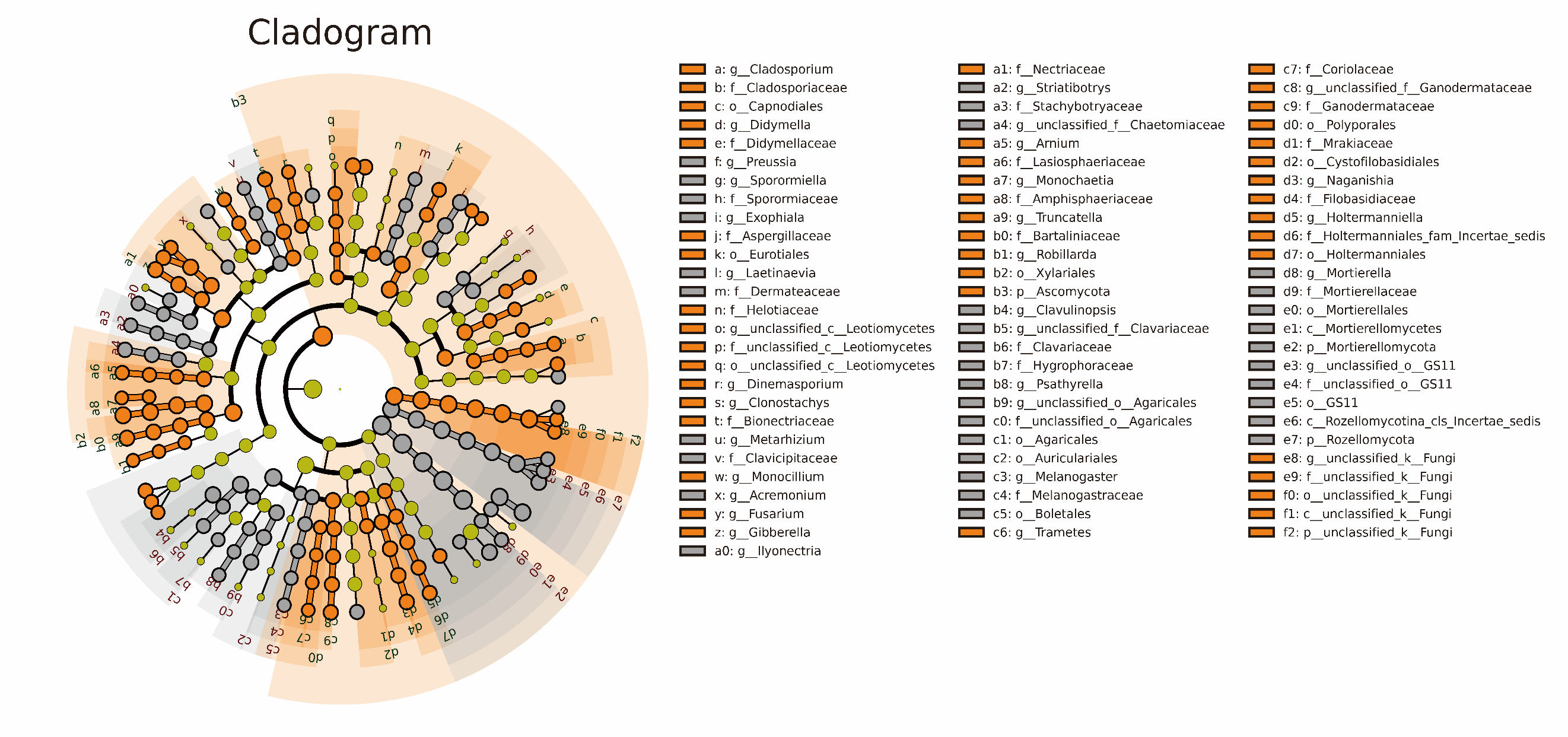


Figure S4 LEfSe analysis detected the key fungi different between licks and the matrix. The circles from the inside to outside indicate fungi from the kingdom to OTU levels. Yellow points represent fungi that did not differ significantly from each other. Orange points and gray points represent fungal biomakers of licks and the matrix, respectively. And most of the insignificant information is not shown in the figure.


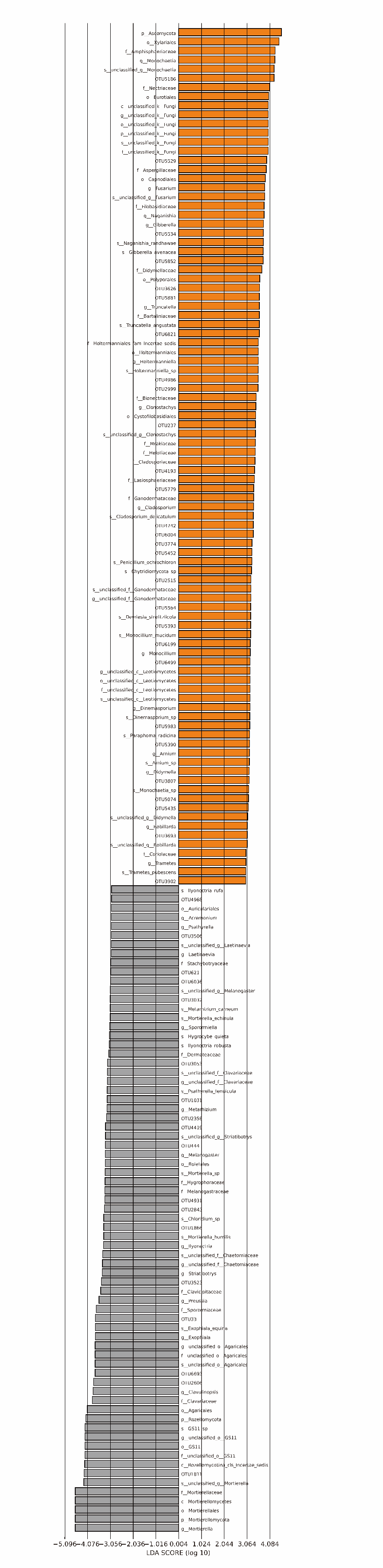


Figure S5 The bar plot shows all marker fungal species (from phylum to OTU) with LDA score greater than 3.0 (p < 0.05). Orange and gray represent fungal biomakers of licks and the matrix, respectively.
